# Supplementary material for: Metagenomic analysis of the intestinal microbiome reveals the potential mechanism involved in Bacillus amyloliquefaciens in treating schistosomiasis japonica in mice
Source: Microbiol Spectr. 2024 Mar 5;12(4):e03735-23. doi: 10.1128/spectrum.03735-23 (PMC10986500; doi:10.1128/spectrum.03735-23)
Supplement: Supplemental material — Fig. S1 and S2. [file spectrum.03735-23-s0001.docx]

**Supplementary Information**


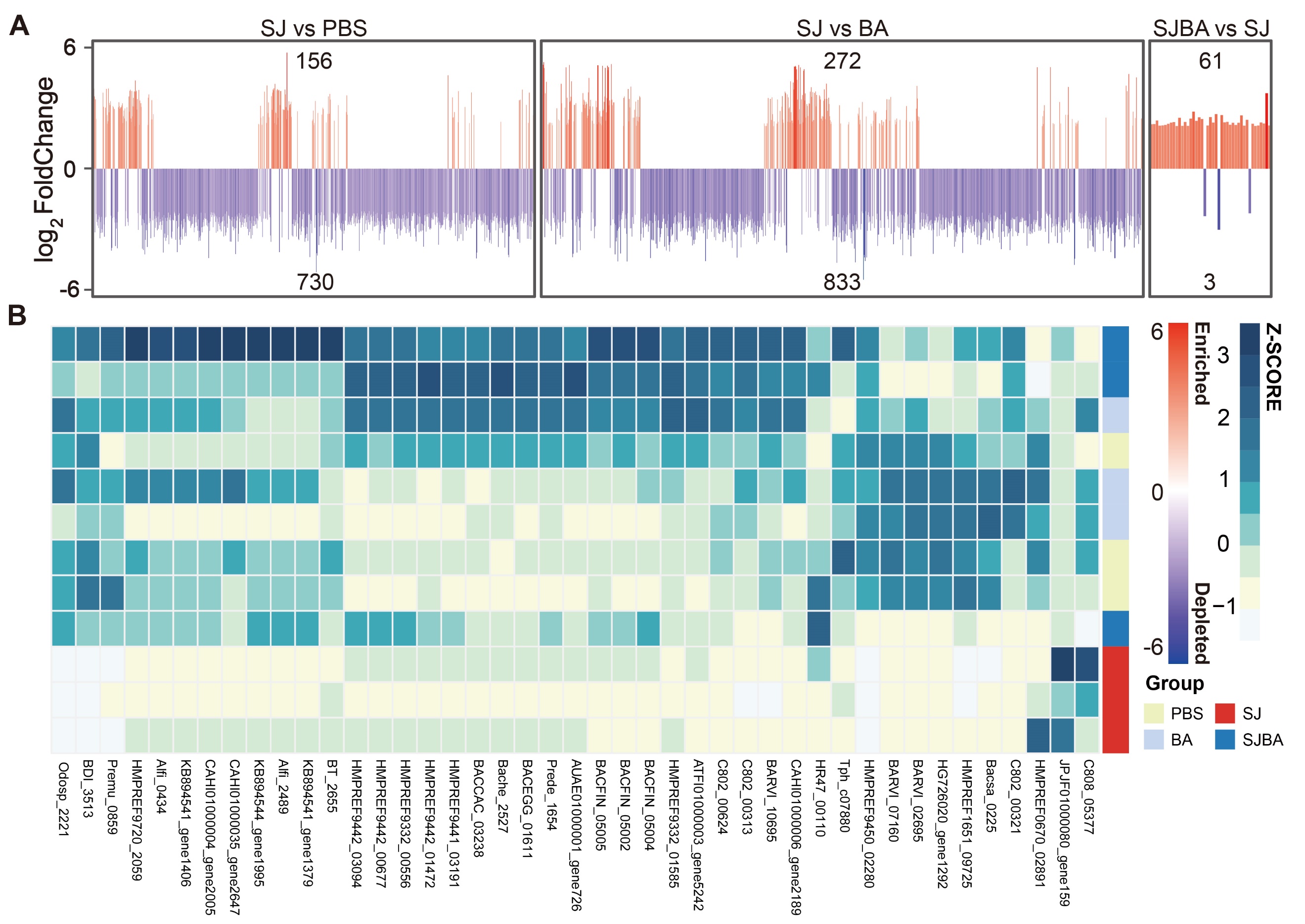


**Figure S1 Screening of genes differentially expressed in abundance. (A)** Screening of genes differentially expressed in abundance. Orange represented genes enriched in expression. Purple represented genes depleted in expression. Numbers meant the number of genes discovered by differential analysis. (B) Heatmap illustrated the abundance of differential genes in four groups.


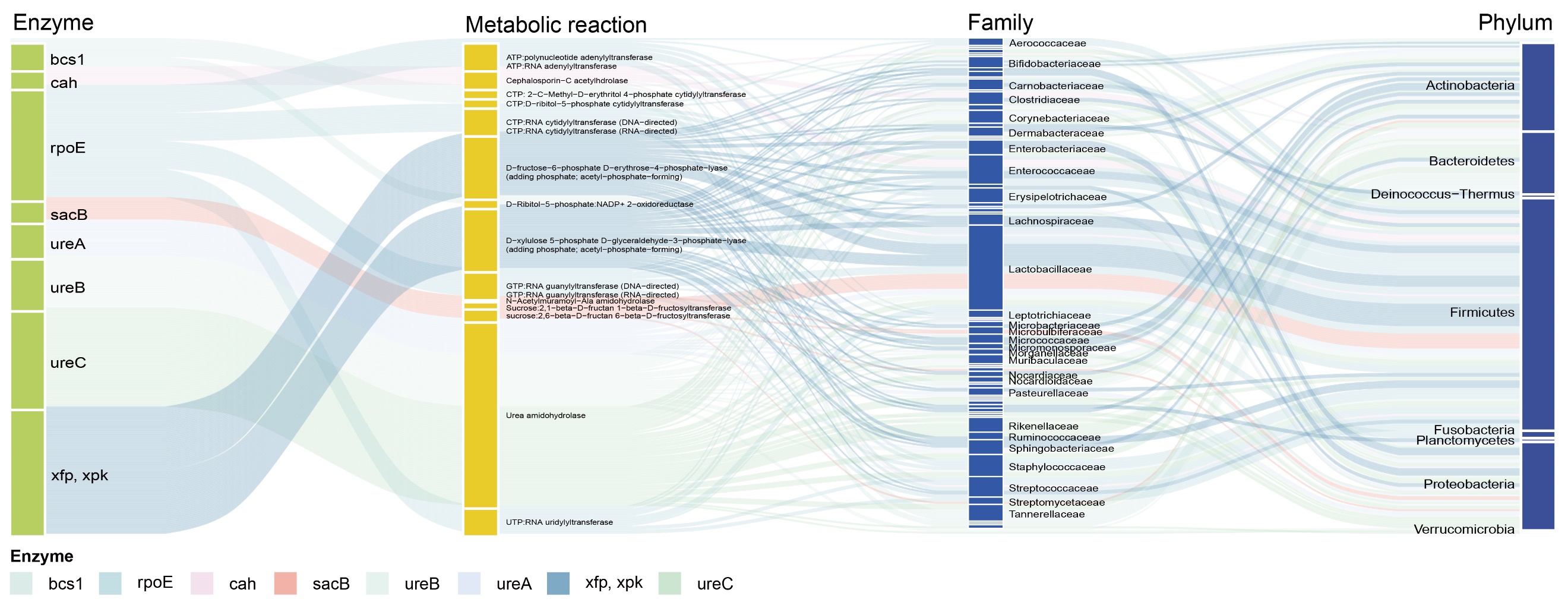


**Figure S2 Association analysis between KEGG database and information of taxonomic classifications.** Sankey plot showed the relationship between KEGG metabolic reactions and taxonomic information which could be linked by nucleotide sequence (Detailed descriptions were presented in the **Results**). Flow zone represented different enzymes.
